# What do animal models tell us about the role of EBV in the pathogenesis of multiple sclerosis?

**DOI:** 10.3389/fimmu.2022.1036155

**Published:** 2022-11-17

**Authors:** Asma Hassani, Gulfaraz Khan

**Affiliations:** ^1^ Dept of Neurology, Division of Movement Disorders, Beth Israel Deaconess Medical Center, Harvard Medical School, Boston, MA, United States; ^2^ Department of Microbiology and Immunology, College of Medicine and Health Sciences, United Arab Emirates University, Al Ain, United Arab Emirates; ^3^ Zayed Center for Health Sciences, United Arab Emirates University, Al Ain, United Arab Emirates

**Keywords:** Epstein-Barr virus, multiple sclerosis, neuroinflammation, animal models, non-human primates (NHP), rodents, rabbits

## Abstract

Multiple sclerosis (MS) is a chronic disease of the central nervous system (CNS), marked primarily by demyelination, inflammation, and neurodegeneration. While the prevalence and incidence rates of MS are on the rise, the etiology of the disease remains enigmatic. Nevertheless, it is widely acknowledged that MS develops in persons who are both genetically predisposed and exposed to a certain set of environmental factors. One of the most plausible environmental culprits is Epstein-Barr virus (EBV), a common herpesvirus asymptomatically carried by more than 90% of the adult population. How EBV induces MS pathogenesis remains unknown. A comprehensive understanding of the biology of EBV infection and how it contributes to dysfunction of the immune system and CNS, requires an appreciation of the viral dynamics within the host. Here, we aim to outline the different animal models, including nonhuman primates (NHP), rodents, and rabbits, that have been used to elucidate the link between EBV and MS. This review particularly focuses on how the disruption in virus-immune interaction plays a role in viral pathogenesis and promotes neuroinflammation. We also summarize the effects of virus titers, age of animals, and route of inoculation on the neuroinvasiveness and neuropathogenic potential of the virus. Reviewing the rich data generated from these animal models could provide directions for future studies aimed to understand the mechanism(s) by which EBV induces MS pathology and insights for the development of prophylactic and therapeutic interventions that could ameliorate the disease.

## Introduction

Multiple sclerosis (MS) is a disease that causes demyelination, or damage to myelin sheaths in the brain, spinal cord and optic nerve (1), leading to disability, particularly among young adults (2). Neuroinflammation is believed to be fundamental to MS pathology (3, 4). T and B lymphocytes infiltrate the central nervous system (CNS) during the early stages of MS, and to a much lesser degree during the late stages (5). Yet, our understanding of what causes pathology in MS is relatively limited.

MS development is believed to be greatly influenced by both genetics and environmental factors. The increased susceptibility to MS in Caucasians compared to other ethnic backgrounds reflects the impact of genes on MS risk (6–8). Among the genes discovered to influence MS risk, HLA class II allele HLADRB1*1501 has been shown to have the greatest odds ratio in conferring susceptibility to MS (9–11). However, the concordance rate for MS is only as high as 25% in genetically identical individuals (12, 13). This implies that other factors are also involved in the development of MS (14). Indeed, immigration studies revealed increased MS risk in immigrants from low MS-risk regions to regions with high MS incidence (15–17). This however, is also impacted by the age at which immigration takes place. Moreover, various environmental factors including infectious agents are thought to modulate susceptibility to MS (18–21).

Substantial evidence from seroepidemiological and pathological studies support the role of Epstein-Barr virus (EBV) infection in MS pathogenesis (22–26). This herpesvirus is one of the most successful pathogens persisting silently in as many as 95% of the human population. It targets circulating B lymphocytes to establish a life-long reservoir in the face of a competent immune surveillance (27, 28). Maintaining a balanced EBV-immune interaction is fundamental to the well-being of the human host. Consequently, several EBV-related pathologies arise in individuals with defects in the immune system (29).

The fact that humans are the only natural host for EBV has made the understanding of how EBV contributes to the development/progression of MS exceptionally challenging. Nevertheless, efforts are continuously made to utilize several animals, including rodents, nonhuman primates (NHP), and rabbits to understand the role of the virus in MS pathogenesis (**Table 1**). Studies have examined the effect of virus titers, age of animals during primary infection, route of inoculation, the direct use of purified virus versus adoptive transfer of virus infected cells, and the induction of experimental autoimmune encephalitis (EAE) in infected versus naïve hosts on the neuroinvasiveness and neuropathogenic potential of the virus. The rich data generated from these animal models has uncovered various mechanisms that viruses, such as EBV potentially use to promote autoimmunity and/or demyelination in the CNS. This review outlines some of the lessons we have learnt from the studies examining the link between EBV and MS using different *in vivo* models.

**Table 1:** Some of the animal models that have been used to examine the link between EBV and MS.

Model	Notable features	Notable drawbacks	Ref
Mice MHV68 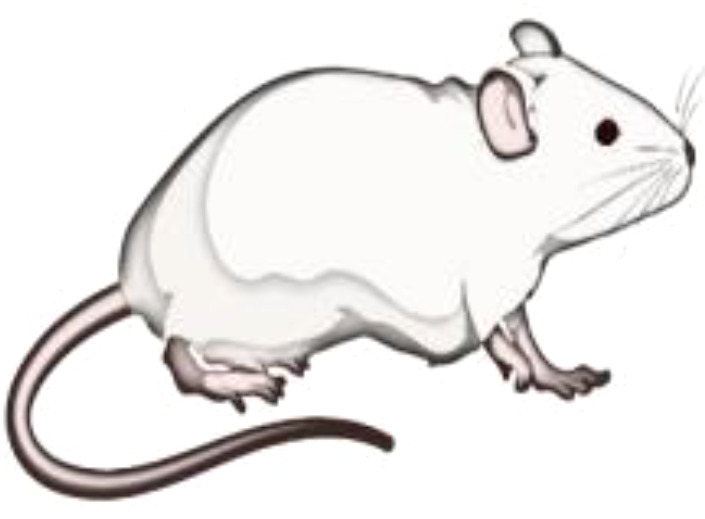	•MHV68 naturally infects rodents and shares several biological characteristics with EBV infection in humans, such as IM. Following primary infection, the virus establishes life-long persistence which can be reactivated upon immunosuppression. • Introduction of high viral dose in the periphery, or directly into the brain of young mice, can lead to CNS infection and associated neuroinflammation which appears to be linked to the expression of viral genes and pro-inflammatory cytokines. • Induction of EAE in mice infected with MHV68 develop more severe disease and is associated with latently infected B-cells.	• MHV68 is significantly different from EBV. LMP and EBNA genes known to be important in EBV- associated pathologies are not present in MHV68. • Additionally, the pathology induced by MHV68 infection does not fully correspond to MS in humans.	(30–38)
Humanized mice 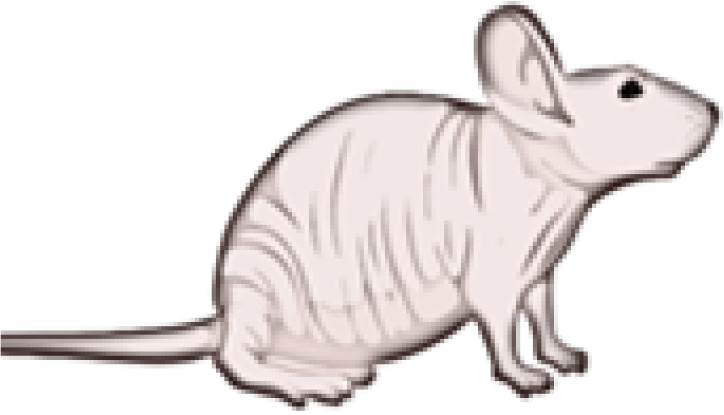	• EBV-infected humanized mice exhibit some pathologies reminiscent of those seen in EBV infection in humans, including IM-like diseases and LPDs. • Humanized mice have been used to explore the biology of EBV in the context of the immune system and genetics. For example, EBV infection of mice reconstituted with HLA-DR15 appear to have poorer control of the virus and are more likely to develop autoimmunity. • Certain types of EBV-infected cells implanted i.v., can traverse the BBB and enter the CNS, leading to neuroinflammation and upregulation of some viral and cellular genes. • When humanized mice are engrafted with PBMCs from EBV-positive or RRMS patients, and then induced to developed EAE, the disease develops earlier with more severe symptoms compared to PBMCs from EBV-negative individuals.	• Normal healthy mice are not susceptible to EBV. • Humanized mice are complex models with many variables due to the changes introduced. Thus, these variables have to be taken into consideration when interpreting the findings using these models.	(39–42)
Rhesus monkeys 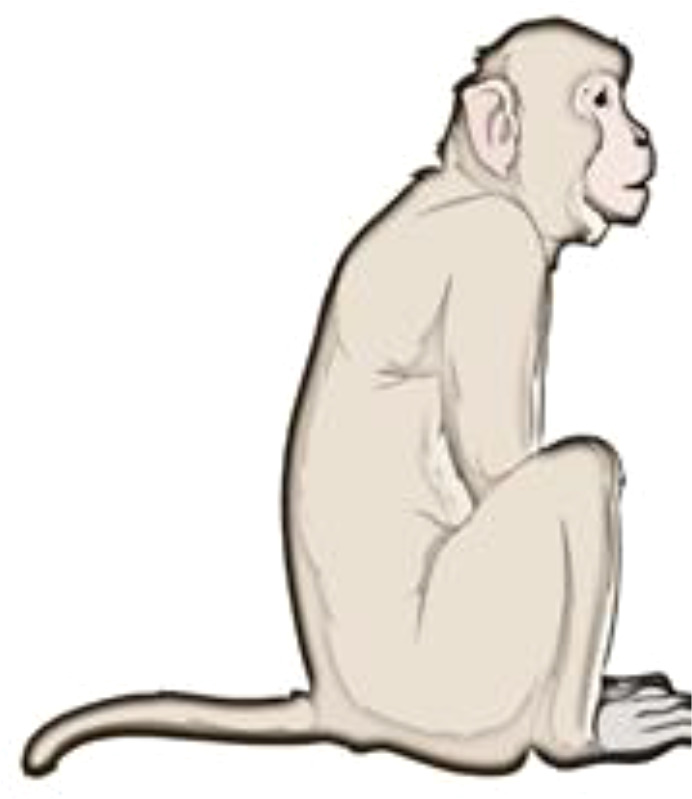	• Rhesus monkeys are natural host for rhLCV. Animals infected with rhLCV reproduce a number of biological features of human EBV infection, including oral shedding and transmission, atypical lymphocytosis, immune response to the virus and long-term persistence. • Animals inoculated with cells immortalized by HVP, a simian α-herpesvirus related to rhLCV, develop neuroinflammation, but no symptomology typically seen in MS. • Neuroinvasion of infected cells into the CNS appears to be associated with both viral as well as immunological factors. For example, blockage of CD28-mediated T-cell costimulation protects animals against EAE.	• rhLCV is a homologue of EBV and hence there are clear differences between the biology and pathogenesis of each virus. • rhLCV is rarely detected in the CNS of infected animals in spite of widespread infection in peripheral organs such as the spleen. • The use of primates is highly restricted and expensive. • Limited centers exist using the primates in medical research.	(43–47)
Japanese macaque 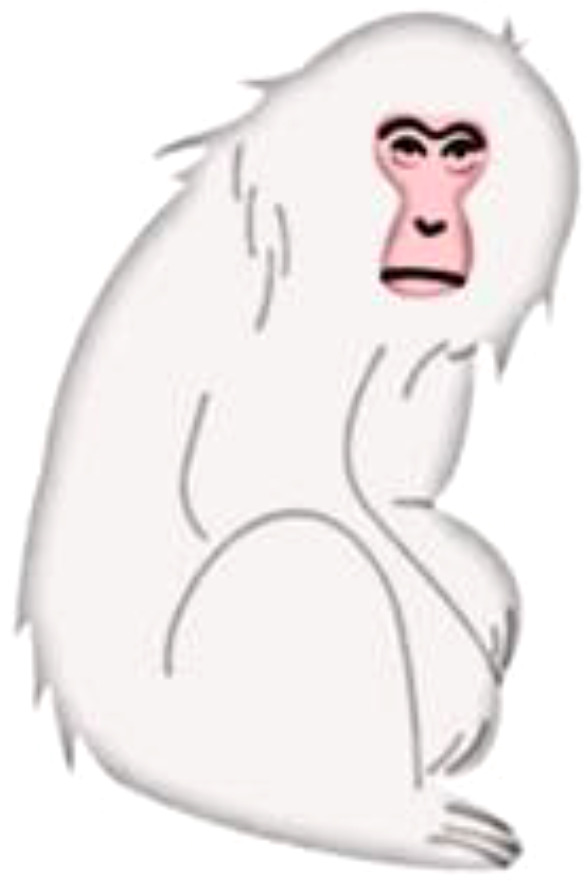	• Japanese macaques have been shown to be natural hosts for JMRV, a γ-herpesvirus with close homology to RRV. • Animals naturally infected with JMRV, spontaneously develop JME, an inflammatory demyelinating disease with clinical and histopathological features resembling MS. A majority of the animals with JME show ataxia, paralysis or paresis of one or more limbs and ocular abnormalities. In most cases the onset of the disease is acute and progresses very rapidly. In contrast to MS, the prevalence of JME is similar in both sexes. • On histopathology, the brain and spinal cord of animals with JME, show acute and chronic multifocal plaque-like demyelinated lesions. Chronic active lesions predominantely consist of macrophages, activated microglia, astrocytes and infiltrating lymphocytes.	• Genetically, JMRV is only distantly related to EBV. Hence, some of the biological and pathological features induced by JMRV can be different from those induced by EBV. • JME develops in macaques spontaneously and the animals often succumb to the disease within about a week following the onset of the symptoms. This makes it difficult to address some pivotal questions in a time-controlled manner. • Medical research on primates is restricted and expensive. • Limited centers exist using the primates in medical research	(48–51)
Rabbit 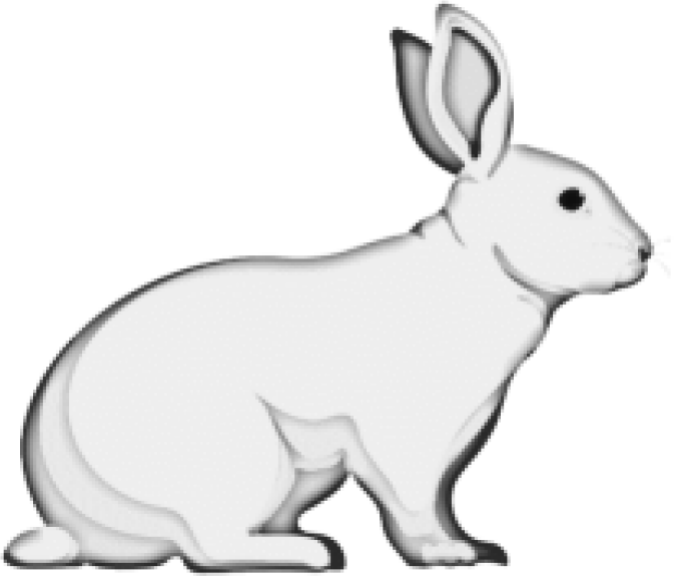	• Rabbits are not natural hosts for EBV but are susceptible to infection upon i.v. inoculation of high doses of the virus. Primary acute infection results in widespread infection, most notable in the spleen. • Infection of healthy immunocompetent rabbits leads to life-long asymptomatic EBV persistence during which little or no virus is detected in PBMCs. Immunosuppression reactivates the virus (type III latency), reminiscent of what has been observed in allograft recipients on immunosuppressive therapy. • Virus-infected cells can also traverse the BBB and enter the CNS, most probably *via* the Trojan horse mechanism involving B-lymphocytes- i.e., infected lymphocytes transport the virus into the CNS parenchyma during the cell influx associated with the inflammatory process. Infected CNS shows distinct inflammatory demyelinating aggregates consisting of blood-derived macrophages, microglia, astrocytes and infiltrating lymphocytes.	• The rabbit model of EBV infection is not a model of MS. Although EBV can traverse the BBB and induces distinct inflammatory changes in the CNS, typical clinical features such as paresis or paralysis are not present. • How the neuroinflammation induced by EBV correlates with MS has not been fully evaluated. • Availability of rabbit-specific reagents is limited, particularly antibodies. This is a major challenge in addressing some of the pivotal questions related to the role of the virus in the pathogenesis of MS.	(52–57)

BBB, Blood-brain barrier; EBNA, Epstein-Barr virus nuclear antigen; EAE, Experimental autoimmune encephalitis; HVP, Herpesvirus papio; IM, Infectious mononucleosis; JME, Japanese macaque encephalomyelitis; JMRV, Japanese macaque rhadinovirus; LMP, Latent membrane protein; LPDs, Lymphoproliferative diseases; MHV68, Murine g-herpesvirus 68; i.v., Intravenous; PBMCs, Peripheral blood mononuclear cells; rhLCV, Rhesus lymphocryptovirus; RRV, Rhesus macaque rhadinovirus.

## EBV and MS in murine models

### Data from Murine γ-herpesvirus 68 infection models

Murine γ-herpesvirus 68 (MHV68, or γHV68) belongs to the gammaherpesvirus subfamily of the Herpesviridae, to which both EBV and human Kaposi’s sarcoma-associated herpesvirus (KSHV, or HHV-8) belong (38). MHV68 is more comparable to KSHV than to EBV in terms of the genomic structure, as both MHV68 and KSHV are gamma-2 viruses while EBV is a gamma-1 virus (38). Nevertheless, MHV68 and EBV share several key biological characteristics during infection of their natural hosts (58) (**Table 1**). For instance, MHV68 infection in mice results in life-long latency in B cells and macrophages, chiefly in the spleen and lungs, with intermittent lytic infection (32, 59, 60). During chronic latent infection, various immune-related genes are differentially altered in several organs including the brain (61). Furthermore, primary MHV68 infection is associated with a short-lived surge in the frequency of activated CD8^+^T cells and splenomegaly in what resembles human infectious mononucleosis (IM) caused by primary EBV infection (33, 62, 63). As a result, several studies utilized MHV68 as a surrogate virus to understand the neuropathogenic potential of EBV, and thus the role of the virus in the pathogenesis of MS.

To determine whether primary peripheral MHV68 infection could be neuroinvasive, Terry and colleagues inoculated various mice strains with MHV68 intranasally (30). Although asymptomatic viral infection was established in the periphery, there was no evidence of viral infection in the CNS. However, peripheral infection was found to cause CNS infection in mice that were deficient in interferon type I receptor (IFNAR), which suggests the protective function of IFNAR against the spread of MHV68 from the periphery to the CNS (30).

The study also examined the consequences of a different route of viral inoculation. When MHV68 was introduced directly into the brain, viral infection was detected in the meninges, ependymal cells, oligodendrocytes, cerebellar Bergmann cells, and pyramidal neuron of the hippocampus. Infection of the brain was also accompanied with infiltration of inflammatory cells into the infected areas and damage to white matter tracts. Viral infection and neuroinflammation were most pronounced towards the end of week 1 post infection (30). These animals exhibited signs of ailment such as lethargy and body atrophy. Nevertheless, inhibiting MHV68 replication in these animals led to silent viral persistence in the CNS. Similarly, introducing non-productively MHV68-infected cells into the brain produced long-term viral persistence in the CNS (30). The ability of MHV68 to establish persistent latent infection in the CNS was also demonstrated by Kang and colleagues (31) following intracerebroventricular inoculation of 9-10wk old BALB/c mice with recombinant MHV68-M3/FL. Viral persistence in the CNS resulted in viral dissemination from the brain to peripheral organs including the spleen and lungs, with the spleen being the main peripheral reservoir for latent MHV68. Immunosuppression with cyclosporin A (CsA) of latently infected mice, after the virus levels had become undetectable in the CNS and periphery, caused virus reactivation, and increased viral load in the brain and spleen (31). While these observations imply that latent infection promote viral persistence in the CNS, the implications of latent virus in the brain of an immunocompetent host are currently unclear.

Additionally, the age of the host can be a crucial factor in determining the outcome of MHV68 infection in the CNS. After intracerebral inoculation, older mice are more likely to survive MHV68 infection than younger mice. Younger mice exhibit a more severe MHV68 infection in the CNS, whereas older mice exhibit a decline in MHV68 load to undetectable levels by the second week of infection (30, 34). MHV68 infection in the CNS has been found to involve increased expression of the proinflammatory cytokines, tumor necrosis factor alpha (TNFα), interleukin 1 beta (IL1β), and IL6 in younger mice (34).

Furthermore, the initial viral load introduced into the periphery influences the neuropathogenic potential of MHV68. In wildtype mice, intranasal inoculation using 2*10^4^ plaque-forming units (pfu) of MHV68 was found to be non-neuroinvasive (30), but inoculating animals with 2*10^5^ pfu of MHV68 intranasally produced detectable and presumably productive infection in the CNS (35). The infection was associated with infiltration of CD45^+^ cells and CD3^+^ cells into different brain areas resulting in meningitis, cerebellitis and perivascular encephalitis. Severe neuroinflammation was more likely to occur in regions of the brain that had increased expression of viral proteins. Over a third of these animals had ataxia, severe dystrophy, or died early (35).

The fact that the risk of MS increases in EBV-infected individuals has raised questions about how a pre-existing infection promotes autoimmune demyelination. Studying EAE in murine models of MHV68 infection revealed that latent virus could be the culprit to exacerbated EAE pathology (36, 64). A study examined myelin oligodendrocyte glycoprotein (MOG)-induced EAE course in C57BL/6 mice 5 weeks post primary infection with MHV68 (i.e., during latent infection) and compared it to EAE course in non-infected animals and animals previously infected with either lymphocytic choriomeningitis virus (LCMV) or murine cytomegalovirus (MCMV) (36). MHV68-infected animals experienced EAE more rapidly, with more severe morbidity and higher risk of mortality than non-infected, LCMV- or MCMV-infected animals. The aggravated disease in MHV68-infected animals appeared to occur in the absence of any signs of productive infection in the CNS (36). This was further corroborated by the observation that mice infected with latency-deficient MHV68 (MHV68 AC-RTA) had an EAE course comparable to that in noninfected controls, indicating that latent infection - rather than acute infection- plays a role in disease progression (64). Indeed, the neuropathological potential of latent infection was also demonstrated by immunizing mice with latent Epstein-Barr virus nuclear antigen 1 amino acid region 411-426 (EBNA-1_411-426_) (65). EBNA-1_411-426_ immunized mice developed neurological signs reminiscent of EAE, and MRI-confirmed cortical lesions (65). This region of EBNA-1 was also found to trigger high antibody response in individuals with relapsing-remitting (RRMS) and secondary progressive MS (SPMS), and these antibodies cross-reacted with myelin-basic protein amino acid region 205-224 (MBP_205-224_). Similarly, mice immunized with EBNA-1_386–405_ in combination with proteolipid protein amino acid region 139-151 (PLP_139–151_) exhibited pronounced EAE course and T helper1 (Th1) phenotype-driven inflammation (66). Notably, immunization with EBNA-1_386–405_ triggered an antibody response against the CNS-derived antigen, glial cell adhesion molecule (GlialCAM). In MS cases, intrathecal antibody response to EBNA-1_386–405_ was found to cross-react with GlialCAM, most probably *via* a molecular mimicry mechanism (66).

EAE pathology in noninfected animals was characterized by infiltration of interferon gamma (IFNγ- and IL17-producing CD4^+^T cells into the spinal cord, whereas MHV68-infected mice had elevated expression of IFNγ and increased infiltration of T bet^+^ CD4 T cells, granzyme B-secreting CD8^+^T cells and F4/80^+^ macrophages/microglia in the brain and spinal cord. Some of the CNS-infiltrating CD8^+^T cells were MHV68-specific. In contrast to noninfected controls, MHV68-infected animals showed demyelination in the cerebellum and corpus callosum (36). Additionally, MHV68-infected mice displayed noticeably greater levels of the costimulatory marker CD40, which appears to promote enhanced expression of the Th1 signature cytokine, IFNγ, and reduction of the frequency of regulatory T cells (Tregs) (64). These results suggest that latent MHV68 infection skews the immune response during EAE toward a Th1 response, rather than a Th17 response, *via* CD40-mediated immune modulation.

How MHV68-infected B cells contribute to EAE pathology was also studied. Animals that received splenic CD19^+^IgD^-^ B cells derived from MHV68-infected animals, before EAE induction, developed severe EAE pathology, including increased production of IFNγ and infiltration of CD8^+^T cells in the brain and spinal cord (37). Antibody-mediated depletion of B cells prior to EAE induction alleviated IFNγ production in the CNS, but was ineffective in eliminating detectable virus in the periphery. On the other hand, B cell depletion prior to primary MHV68 infection led to EAE disease similar to that in control animals that received noninfected B cells (37). This implies that B cells are fundamental for MHV68 to establish latency, which in turn contributes to the exacerbation of EAE disease. EAE was also observed to be aggravated in mice that received EBV-immortalized B lymphoblastoid cell lines (BLCLs) derived from patients with SPMS, prior to MOG-EAE induction (67). Furthermore, mice that were given these BLCLs and recovered from the initial EAE course, experienced disease relapses. These animals also exhibited marked changes in the makeup of their gut microbiota (67). This may shed insight into potential mechanisms, by which EBV-infected and transformed B cells could aggravate autoimmunity by interfering in otherwise balanced gut-brain axis.

### Data from humanized mice

Humanized mice have also been utilized to examine EBV involvement in MS (**Table 1**). Humanized mice, such as NOD SCID IL2Rγ- deficient (NSG) mice, are immunodeficient animals that can be successfully engrafted with human hematopoietic stem cells to rebuild functional parts of the human immune system. Studies in humanized mice can help delineate how strictly human pathogens such as EBV, behave in their natural host. EBV infection in humanized mice can result in acute IM-like disease, latent infection, or lymphoproliferative disorders (LPDs), depending on the dose of the inoculum (39, 68, 69).

A recent study explored the association between EBV infection and HLA-DR15 in NSG mice (41) in light of the observation that individuals with a history of IM and carrying the HLA-DRB1*15:01 allele are more likely to develop MS (70). Mice reconstituted with human HLA-DR15 hematopoietic progenitor cells displayed significantly greater numbers of activated CD8^+^T cells in the periphery (blood and spleen), at 4-6 weeks post EBV infection, compared to controls. EBV-infected mice reconstituted with either HLA-DR15-negative or HLA-DR4-positive immune compartments served as controls. EBV load was also greater in EBV-infected HLA-DR15-carrying animals than in controls. EBV load correlated positively with frequencies and total numbers of activated CD8^+^T cells in the periphery. Thus, the genetic risk associated with HLA-DR15 may mediate impaired control over EBV infection, and likely contributes to the pathogenesis of IM (41). The study also demonstrated that CD4^+^T cells derived from HLA-DR15-carrying mice can recognize MBP epitopes and allogeneic targets. HLA-DR15-restricted cells were found to respond more robustly and non-specifically to HLA-mismatched targets than to their HLA-matched targets. This cross-reactivity mechanism, in conjunction with the lack of specificity in the T cell response, may explain how EBV and the HLA-DR15 allele synergistically enhance MS risk (41).

Another study used NSG mice to assess the impact of EBV on EAE course. NSG mice were transplanted with PBMCs isolated from either patients with relapsing-remitting MS (RRMS), EBV-seropositive healthy controls, or EBV- seronegative healthy controls (42). Upon induction of EAE, all mice exhibited EAE signs. Nonetheless, mice engrafted with RRMS PBMCs showed a more pronounced disease course than mice engrafted with non-MS PBMCs. Furthermore, mice engrafted with PBMCs from EBV seropositive donors had more rapid onset of EAE than animals reconstituted with PBMCs from EBV seronegative donors (42). Hence, this model emphasizes the fundamental role of EBV in disease progression.

Studies on NSG mice have also helped us understand factors that promote migration of EBV-infected cells from the periphery to the brain. NSG mice implanted intravenously, intracardially or subcutaneously with the MUN14 cell line derived from an EBV+ Burkitt’s lymphoma, showed increased trafficking of EBV-infected cells to the brain (71). As a result, mice developed neurological signs including gait deficits, tremor and seizures. Epigenetic changes in EBV-infected B cells were found to enhance the neuroinvasiveness of infected cells. Increased expression of viral protein EBNA-1, and cellular phosphoprotein-1/osteopontin were suspected to be involved in driving the epigenetic changes leading to the neuroinvasive phenotype (71).

## Nonhuman primate model for EBV infection of the brain

### Lymphocryptovirus-infected B cells

Nonhuman primates (NHPs) have also served as an appealing model to better understand how EBV modulates the risk of MS (**Table 1**). A study on adult rhesus monkeys (Macaca mulatta) examined whether EBV-infected and transformed B cells can (1) present myelin antigen to, and activate myelin-specific T cells, and (2) promote inflammation in the brain (47). Herpesvirus papio (HVP), a baboon-tropic virus that shares biological and genetic similarities with EBV, was used to infect and transform B cells isolated from rhesus monkeys and subsequently generate BLCLs. These cells were then loaded with either MOG_34-56_, citrullinated MOG_34-56_, or CMV capsid antigen-derived peptides, and infused back into the monkeys. Animals exhibited only weight loss with no signs of neurological deficits. However, they mounted an immune response made up primarily of CD8^+^T cells, CD8^+^CD56^+^T cells, and CD3^-^CD56^+^NK cells against MOG and viral peptides (47). Interestingly, myelin-specific T cells reacted against MOG only when its peptides were presented by BLCLs. Moreover, animals, particularly those infused with auto-BLCLs pulsed with citrullinated MOG_34-56_ exhibited meningeal inflammation and infiltration of T cells, B cells, and macrophages into the brain, with no major signs of myelin destruction (47). These observations point to the pathogenic role of EBV-infected and transformed B cells in neuroinflammation by serving as efficient APCs and promoting recognition of and response against myelin antigens by myelin-specific T cells. This pathogenic effect of EBV-infected B cells was also demonstrated in another NHP, the marmoset (Callithrix jacchus) (72). Jagessar and coathors proposed an explanation for this. EBV infection of B cells enables productive processing of myelin peptides for presentation to autoreactive T cells (73). B cells in absence of the influence of EBV infection would instead abort antigen processing and opt for the degradation of self-antigen (MOG_34-56_) which ultimately prevent the presentation of MOG_34-56_ to their autoreactive T cells (74).

### Japanese macaque rhadinovirus and spontaneous onset of encephalomyelitis

NHPs have also emerged as a natural model for herpesvirus-associated autoimmune demyelinating diseases. Japanese macaques (Macaca fuscata) housed at the Oregon National Primate Research Center have been found to naturally develop Japanese macaque encephalomyelitis (JME), an MS-like disease, at a rate of 1-3% annually since 1986 (49). In contrast to MS where females are at higher risk than males, JME is believed to develop in both sexes at comparable rates, with a reported median age at disease onset of ~4.3 years. Furthermore, the majority of animals experience acute neurological signs that are almost immediately followed by rapid progression that necessitates euthanasia within a median time of 6 days. Neurological signs commonly include ataxia, paralysis or paresis of at least one limb, and less commonly ocular paresis, body tremors, and head tilt (49). Antemortem MRI examination of the CNS, using post-gadolinium contrast T_1_-weighted images and T_2_-weighted axial images of affected animals showed several conspicuous lesions in the white matter of both the brain and spinal cord. Pathologically, these lesions were restricted to the white matter and characterized by demyelination, disrupted axons and marked influx of immune cells in what resembles chronic active MS plaques (49). Unlike MS, pathological changes in the meninges and cortical grey matter are not characteristics of JME (49). JME white matter lesions are characterized by the infiltration of CD163^+^ cells, some of which are MBP-reactive, the aggregation of CD20^+^B cells in periventricular areas, and the presence of CD4^+^T cells, CD8^+^T cells, and Th17 cells at variable levels (51). Similar to MS patients, JME-affected animals produce intrathecal oligoclonal bands (51).

Interestingly, a newly characterized gamma-2 herpesvirus was recovered from these lesions (48, 49). The virus termed Japanese macaque rhadinovirus (JMRV) had 89.5% and 47.9% sequence homology with rhesus macaque rhadinovirus and KSHV, respectively (49). However, JMRV does not appear to share considerable genetic homology with EBV (48). Subsequent studies described the complete sequence of the viral genome, which revealed both conserved and unique open-reading frames (ORFs), and viral-encoded miRNAs that may be implicated in disease pathogenesis (48, 75).

In spite of the presence of the virus in JME lesions, the intrathecal humoral response does not appear to be directed against the virus (50). T lymphocytes specific for MBP, MOG, and PLP infiltrate the brain and circulate in peripheral blood of infected animals (50).

## Rabbit model of EBV infection

### Rabbits are susceptible to EBV infection

A number of reports have shown that New Zealand White (NZW) rabbits are susceptible to EBV, and the infection mimics that observed in humans (52–55, 76) (**Table 1**). During primary EBV infection, rabbits show no symptoms, but can experience temporarily enlarged lymph nodes and spleens, short-term lymphocytosis, elevated titers of anti-EBV viral capsid antigen antibodies, and detectable levels of EBV genome within the first 2 weeks of infection (57). However, EBV levels vary between different rabbits, and fluctuate overtime in a given animal (57). This suggests that different infection dynamics are influenced by differences in rabbit immune responses to EBV, similar to those observed in humans. Similar to humans, the rabbit immune system does not completely eradicate EBV infection, as EBV can persist latently in these animals (52). EBV infection in rabbits produces a robust humoral response, which helps reduce viral loads below detectable levels in the blood, whereas cyclosporin A (CsA)-mediated suppression of T cells can cause reactivation of the latent virus (53).

### Peripheral EBV infection of rabbits can lead to CNS infection

A recent study examined if EBV, following primary infection, could enter the rabbit CNS and by what mechanism (56). The study revealed that, at day 14 of infection, the levels of viral DNA were highest in both peripheral and CNS compartments. Although viremia was pronounced at day 14 of infection, EBV load in the brain did not correlate with the levels of free virus in the circulation. There was, however, a positive correlation between the levels of cell-associated virus (i.e., infected cells) in the periphery and EBV levels in the brain. Thus, we posit that migrating infected cells, presumably B lymphocytes, could be the primary source of EBV infection in the brain (77). Indeed, some brain infiltrating B lymphocytes were infected with EBV, in addition to a few infected astrocytes and microglia.

### EBV infection of the CNS induces the formation of inflammatory cellular aggregates

**Figure 1:**
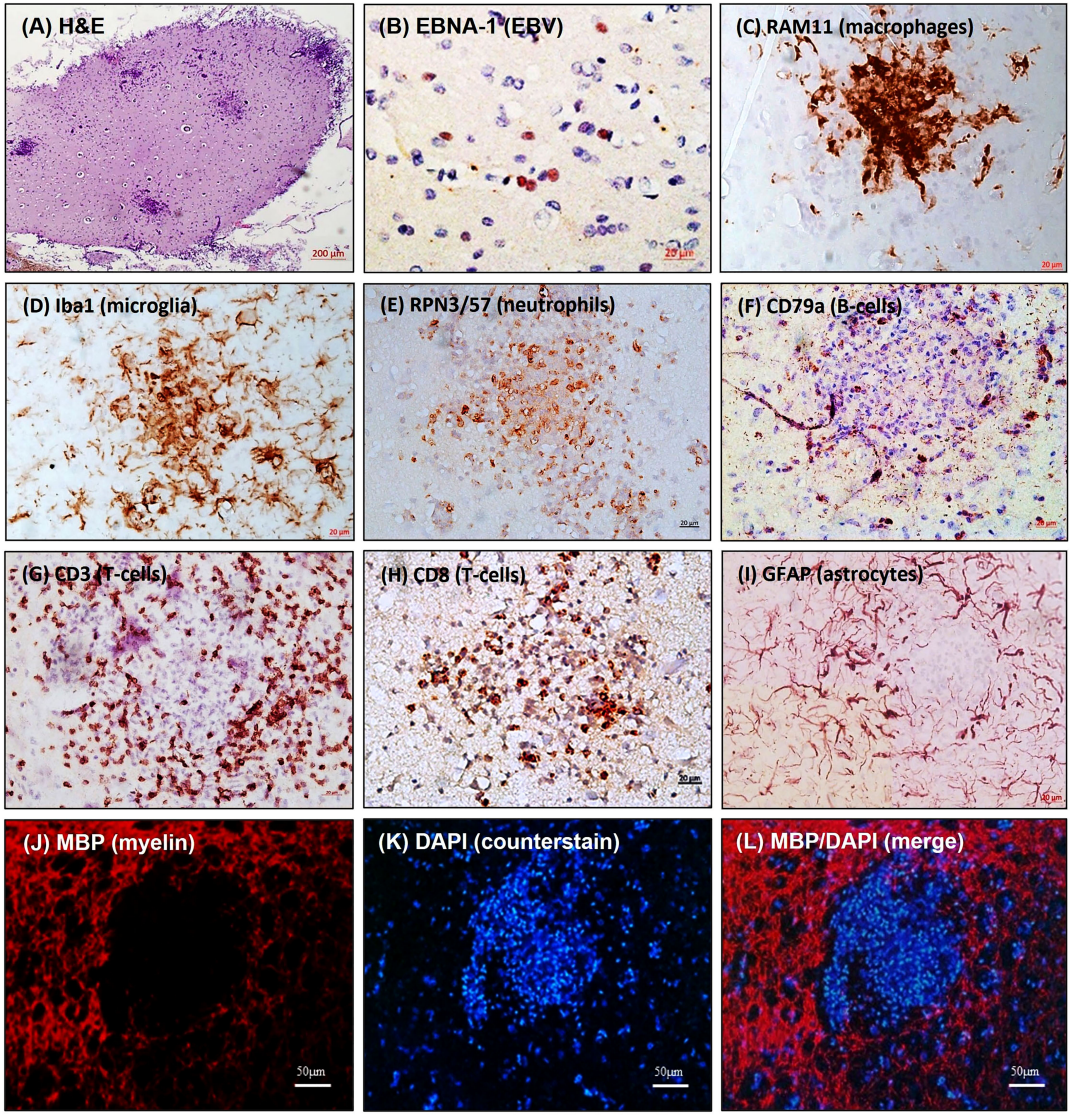
Inflammatory cellular aggregates in brain of EBV-infected rabbits. Brain sections were stained for: **(A)** H&E; **(B)** EBV latent nuclear protein, EBNA-1; **(C)** Blood-derived macrophage marker, RAM11; **(D)** Microglia marker, Iba1; **(E)** Neutrophil marker, RPN3/57; **(F)** B-cell marker, CD79a; **(G)** pan-T-cell marker, CD3; **(H)** Cytotoxic T-cell marker, CD8; **(I)** Astrocyte marker, GFAP; **(J)** Myelin marker, MBP; **(K)** Counter stain, DAPI; **(L)** MBP/DAPI merge showing areas of demyelination [adapted from (56)]. The figure shows non-serial sections from the same block. (Scale bar= 200µm in **A**, 20µm in **B–I**, and 50µm in **J–L**).

Primary EBV infection has been investigated in healthy rabbits and rabbits immunosuppressed with cyclosporine A (CsA). In line with previous data, EBV load increased significantly when the immune system was suppressed, and this raised the likelihood of virus-infected cells breaching CNS barriers. Despite the absence of overt signs of neurological impairments, peripheral EBV infection induced neuroinflammatory cellular aggregates in some animals within 2 weeks of infection (**Figure 1**). These animals displayed focal CNS cellular aggregates composed of densely clustered blood-derived macrophages surrounded by activated microglia and astrocytes, neutrophils, CD8^+^ T lymphocytes, dispersed EBI2^+^ cells and PCNA^+^, IgM^+^, and IgG^+^ B lymphocytes (**Figure 1**). Remarkably, the cell aggregates showed myelin damage in the center (**Figure 1**) (56). This may model the early stages of lesions that progress into MS smoldering active plaques (78). Of interest, some of the immunosuppressed noninfected controls also developed cell aggregates in the CNS, which was probably brought on by the reactivation of opportunistic intrinsic pathogens. This scenario mimics the development of CNS pathology seen in immunosuppressed individuals who experience progressive multifocal leukoencephalopathy as a result of JC virus reactivation (79). Nonetheless, the distinctions between neuroinflammation induced by EBV infection and that attributed to immunosuppression warrants further investigation. Similarly, further investigations are required to understand how the cellular aggregates seen in the rabbits correlates with ectopic lymphoid follicles reported in the brain of MS patients (4, 80, 81).

### Expression of EBV latent transcripts correlates with proinflammatory cytokines

Importantly, EBV latent transcripts, most notably EBV-encoded RNAs (EBERs) correlate positively with the proinflammatory cytokines IL1β and IL6 in the brain and spleen (56). EBER2 expression *in vitro* is associated with high levels of IL-6 produced by infected B cells (82). This proinflammatory cytokine appears to be instrumental for the activation and expansion of EBV-infected B cells (82, 83). Additionally, proinflammatory IL-6 is markedly elevated in rabbits developing aggregates in the CNS. Thus, it is possible that this cytokine is a major player in the pathogenesis of EBV-associated neuroinflammation.

The mRNA levels of IL-1β, IFN-γ and TNF-α have also been observed to be significantly elevated at day 28 of infection in the spleen, brain and the spinal cord of rabbits. The expression of these cytokines in the CNS is associated with impairment of the brain-blood barrier (BBB) (84–88). CNS viral infections can trigger the production of these inflammatory cytokines, which compromises the integrity of BBB, for example by altering the expression of brain endothelia tight junction proteins (89–92). Thus, BBB breakdown could be both a pre-requisite and a consequence of CNS viral infections (90, 91, 93). One could argue that increased mRNA levels of these cytokines in EBV-infected rabbits may induce BBB leakage and recurrent influx of immune cells into the CNS. Whether EBV infection disrupts BBB integrity warrants further investigation.

## Concluding remarks

In spite of substantial efforts over the last 6 decades in studying EBV, there are still many important gaps in our understanding of the details of viral pathogenesis and key aspects of the virus life cycle. There is a pressing need to understand how the virus behaves in the host and how that affects various organ systems. Studies on the link between EBV infection and the pathogenesis of CNS diseases, including MS is enormously expanding. There is now substantial and credible evidence that EBV is involved in the pathogenesis of MS (26, 66, 94, 95). Importantly, a number of studies have demonstrated the presence of EBV-infected cells directly in the white matter lesions in MS tissues (96–99). However, the mechanism by which EBV induces MS remains unclear. To characterize viral dynamics and virus interaction with the immune system, host genetic background and environmental cofactors, it is necessary to develop an *in vivo* model that captures both EBV biology and MS pathology. This review highlights the lessons we have learnt from several animal models used to understand the link between EBV and MS. While none of the models discussed here is a perfect representation of “EBV-induced MS”, utilizing them has provided insight into a number of potential factors, such as viral latent cycle and viral latent proteins, EBV-infected and transformed B cells, HLA-DR15, and epigenetic regulation of EBV-infected B cells, that may contribute to virus-induced CNS pathology. Exploring the various *in vivo* models for EBV would not only aid in the early identification of at-risk populations but also provide promising possibilities for therapeutic and prophylactic options for this incurable disease.

## Author contributions

Conceptualization, writing the initial draft, and revision was performed by AH; conceptualization, writing part of the first draft and revision was performed by GK. All authors contributed to the article and approved the submitted version.

## Funding

This work was funded by UAEU Zayed Centre-Based grants 31R259.

## Acknowledgments

We would like to thank the staff in the Animal House, UAE University, College of Medicine and Health Sciences for their continuous support in helping and looking after the rabbits used in the original studies cited here.

## Conflict of interest

The authors declare that the research was conducted in the absence of any commercial or financial relationships that could be construed as a potential conflict of interest.

## Publisher’s note

All claims expressed in this article are solely those of the authors and do not necessarily represent those of their affiliated organizations, or those of the publisher, the editors and the reviewers. Any product that may be evaluated in this article, or claim that may be made by its manufacturer, is not guaranteed or endorsed by the publisher.

## References

[1] Reich DS Lucchinetti CF Calabresi PA. Multiple sclerosis. N Engl J Med (2018) 378:169–80. doi: 10.1056/NEJMra1401483 PMC6942519 29320652

[2] Dobson R Giovannoni G. Multiple sclerosis - a review. Eur J Neurol (2019) 26:27–40. doi: 10.1111/ene.13819 30300457

[3] Bevan RJ Evans R Griffiths L Watkins LM Rees MI Magliozzi R. Meningeal inflammation and cortical demyelination in acute multiple sclerosis. Ann Neurol (2018) 84:829–42. doi: 10.1002/ana.25365 30362156

[4] Pikor NB Prat A Bar-Or A Gommerman JL. Meningeal tertiary lymphoid tissues and multiple sclerosis: A gathering place for diverse types of immune cells during CNS autoimmunity. Front Immunol (2016) 6:657. doi: 10.3389/fimmu.2015.00657 26793195 PMC4710700

[5] Lassmann H. Multiple sclerosis pathology. Cold Spring Harb Perspect Med (2018) 8:a028936. doi: 10.1101/cshperspect.a028936 29358320 PMC5830904

[6] Brum DG Luizon MR Santos AC Lana-Peixoto MA Rocha CF Brito ML. European Ancestry predominates in neuromyelitis optica and multiple sclerosis patients from Brazil. PloS One (2013) 8:e58925. doi: 10.1371/journal.pone.0058925 23527051 PMC3604018

[7] Flores J González S Morales X Yescas P Ochoa A Corona T. Absence of multiple sclerosis and demyelinating diseases among lacandonians, a pure Amerindian ethnic group in Mexico. Multiple Sclerosis Int (2012) 2012:e292631. doi: 10.1155/2012/292631 PMC3437645 22973516

[8] Hammond SR de Wytt C Maxwell IC Landy PJ English D McLeod JG. The epidemiology of multiple sclerosis in Queensland, Australia. J Neurological Sci (1987) 80:185–204. doi: 10.1016/0022-510X(87)90154-7 3681330

[9] Barcellos LF Sawcer S Ramsay PP Baranzini SE Thomson G Briggs F. Heterogeneity at the HLA-DRB1 locus and risk for multiple sclerosis. Hum Mol Genet (2006) 15:2813–24. doi: 10.1093/hmg/ddl223 16905561

[10] Chao MJ Barnardo MCNM Lincoln MR Ramagopalan SV Herrera BM Dyment DA. HLA class I alleles tag HLA-DRB1*1501 haplotypes for differential risk in multiple sclerosis susceptibility. Proc Natl Acad Sci U.S.A. (2008) 105:13069–74. doi: 10.1073/pnas.0801042105 PMC2529028 18765817

[11] Ramagopalan SV Ebers GC. Multiple sclerosis: major histocompatibility complexity and antigen presentation. Genome Med (2009) 1:105. doi: 10.1186/gm105 19895714 PMC2808740

[12] French Research Group on Multiple Sclerosis Multiple sclerosis in 54 twinships: concordance rate is independent of zygosity. French research group on multiple sclerosis. Ann Neurol (1992) 32:724–7. doi: 10.1002/ana.410320604 1471862

[13] Willer CJ Dyment DA Risch NJ Sadovnick AD Ebers GC Canadian Collaborative Study Group. Twin concordance and sibling recurrence rates in multiple sclerosis. PNAS (2003) 100:12877–82. doi: 10.1073/pnas.1932604100 PMC240712 14569025

[14] Fagnani C Neale MC Nisticò L Stazi MA Ricigliano VA Buscarinu MC. Twin studies in multiple sclerosis: A meta-estimation of heritability and environmentality. Mult Scler (2015) 21:1404–13. doi: 10.1177/1352458514564492 25583848

[15] Berg-Hansen P Moen SM Sandvik L Harbo HF Bakken IJ Stoltenberg C. Prevalence of multiple sclerosis among immigrants in Norway. Mult Scler (2015) 21:695–702. doi: 10.1177/1352458514554055 25344371

[16] Berg-Hansen P Celius EG. Socio-economic factors and immigrant population studies of multiple sclerosis. Acta Neurol Scand (2015) 132:37–41. doi: 10.1111/ane.12429 26046557

[17] Nasr Z Majed M Rostami A Sahraian MA Minagar A Amini A. Prevalence of multiple sclerosis in Iranian emigrants: review of the evidence. Neurol Sci (2016) 37:1759–63. doi: 10.1007/s10072-016-2641-7 27351545

[18] Alharbi FM. Update in vitamin d and multiple sclerosis. Neurosci (Riyadh) (2015) 20:329–35. doi: 10.17712/nsj.2015.4.20150357 PMC4727614 26492110

[19] Hedström AK Hillert J Olsson T Alfredsson L. Smoking and multiple sclerosis susceptibility. Eur J Epidemiol (2013) 28:867–74. doi: 10.1007/s10654-013-9853-4 PMC3898140 24146047

[20] Libbey JE Cusick MF Fujinami RS. Role of pathogens in multiple sclerosis. Int Rev Immunol (2014) 33:266–83. doi: 10.3109/08830185.2013.823422 PMC4369909 24266364

[21] Munger KL Chitnis T Ascherio A. Body size and risk of MS in two cohorts of US women. Neurology (2009) 73:1543–50. doi: 10.1212/WNL.0b013e3181c0d6e0 PMC2777074 19901245

[22] Ascherio A Munger KL Lennette ET Spiegelman D Hernán MA Olek MJ. Epstein-Barr Virus antibodies and risk of multiple sclerosis: a prospective study. JAMA (2001) 286:3083–8. doi: 10.1001/jama.286.24.3083 11754673

[23] Ascherio A Munger KL. 99th dahlem conference on infection, inflammation and chronic inflammatory disorders: Epstein–Barr virus and multiple sclerosis: epidemiological evidence. Clin Exp Immunol (2010) 160:120–4. doi: 10.1111/j.1365-2249.2010.04121.x PMC2841845 20415861

[24] Belbasis L Bellou V Evangelou E Ioannidis JPA Tzoulaki I. Environmental risk factors and multiple sclerosis: an umbrella review of systematic reviews and meta-analyses. Lancet Neurol (2015) 14:263–73. doi: 10.1016/S1474-4422(14)70267-4 25662901

[25] Giovannoni G Cutter GR Lunemann J Martin R Münz C Sriram S. Infectious causes of multiple sclerosis. Lancet Neurol (2006) 5:887–94. doi: 10.1016/S1474-4422(06)70577-4 16987736

[26] Bjornevik K Cortese M Healy BC Kuhle J Mina MJ Leng Y. Longitudinal analysis reveals high prevalence of Epstein-Barr virus associated with multiple sclerosis. Science (2022) 375:296–301. doi: 10.1126/science.abj8222 35025605

[27] Cohen JI. Herpesvirus latency. J Clin Invest (2020) 130:3361–9. doi: 10.1172/JCI136225 PMC7324166 32364538

[28] Young LS Yap LF Murray PG. Epstein-Barr Virus: more than 50 years old and still providing surprises. Nat Rev Cancer (2016) 16:789–802. doi: 10.1038/nrc.2016.92 27687982

[29] Taylor GS Long HM Brooks JM Rickinson AB Hislop AD. The immunology of Epstein-Barr virus-induced disease. Annu Rev Immunol (2015) 33:787–821. doi: 10.1146/annurev-immunol-032414-112326 25706097

[30] Terry LA Stewart JP Nash AA Fazakerley JK. Murine gammaherpesvirus-68 infection of and persistence in the central nervous system. J Gen Virol (2000) 81:2635–43. doi: 10.1099/0022-1317-81-11-2635 11038374

[31] Kang H-R Cho H-J Kim S Song IH Lee TS Hwang S. Persistent infection of a gammaherpesvirus in the central nervous system. Virology (2012) 423:23–9. doi: 10.1016/j.virol.2011.11.012 22169075

[32] Sunil-Chandra NP Efstathiou S Nash AA. Murine gammaherpesvirus 68 establishes a latent infection in mouse b lymphocytes in vivo. J Gen Virol (1992) 73(Pt 12):3275–9. doi: 10.1099/0022-1317-73-12-3275 1469366

[33] Sunil-Chandra NP Efstathiou S Arno J Nash AA. Virological and pathological features of mice infected with murine gamma-herpesvirus 68. J Gen Virol (1992) 73(Pt 9):2347–56. doi: 10.1099/0022-1317-73-9-2347 1328491

[34] Cho H-J Kim S Kwak S-E Kang T-C Kim H-S Kwon H-J. Age-dependent pathogenesis of murine gammaherpesvirus 68 infection of the central nervous system. Mol Cells (2009) 27:105–11. doi: 10.1007/s10059-009-0011-5 19214440

[35] Häusler M Sellhaus B Scheithauer S Engler M Alberg E Teubner A. Murine gammaherpesvirus-68 infection of mice: A new model for human cerebral Epstein–Barr virus infection. Ann Neurol (2005) 57:600–3. doi: 10.1002/ana.20440 15786475

[36] Casiraghi C Shanina I Cho S Freeman ML Blackman MA Horwitz MS. Gammaherpesvirus latency accentuates EAE pathogenesis: Relevance to Epstein-Barr virus and multiple sclerosis. PloS Pathog (2012) 8:e1002715. doi: 10.1371/journal.ppat.1002715 22615572 PMC3355105

[37] Márquez AC Shanina I Horwitz MS. Multiple sclerosis-like symptoms in mice are driven by latent γHerpesvirus-68 infected b cells. Front Immunol (2020) 11:584297. doi: 10.3389/fimmu.2020.584297 33329556 PMC7711133

[38] Virgin HW Latreille P Wamsley P Hallsworth K Weck KE Dal Canto AJ. Complete sequence and genomic analysis of murine gammaherpesvirus 68. J Virol (1997) 71:5894–904. doi: 10.1128/JVI.71.8.5894-5904.1997 PMC191845 9223479

[39] Fujiwara S Imadome K-I Takei M. Modeling EBV infection and pathogenesis in new-generation humanized mice. Exp Mol Med (2015) 47:e135–5. doi: 10.1038/emm.2014.88 PMC4314584 25613732

[40] Traggiai E Chicha L Mazzucchelli L Bronz L Piffaretti J-C Lanzavecchia A. Development of a human adaptive immune system in cord blood cell-transplanted mice. Science (2004) 304:104–7. doi: 10.1126/science.1093933 15064419

[41] Zdimerova H Murer A Engelmann C Raykova A Deng Y Gujer C. Attenuated immune control of Epstein-Barr virus in humanized mice is associated with the multiple sclerosis risk factor HLA-DR15. Eur J Immunol (2021) 51:64–75. doi: 10.1002/eji.202048655 32949466

[42] Allanach JR Hardman BK Fettig NM Mouat I Gu Y Jean-Baptiste V. Insights into the role of Epstein-Barr virus infection in multiple sclerosis using a novel humanized mouse model of disease. J Immunol (2020) 204:58.9–9.

[43] Moghaddam A Rosenzweig M Lee-Parritz D Annis B Johnson RP Wang F. An animal model for acute and persistent Epstein-Barr virus infection. Science (1997) 276:2030–3. doi: 10.1126/science.276.5321.2030 9197263

[44] Fujiwara S Nakamura H. Animal models for gammaherpesvirus infections: Recent development in the analysis of virus-induced pathogenesis. Pathogens (2020) 9(2):116. doi: 10.3390/pathogens9020116 32059472 PMC7167833

[45] Tyler SD Severini A. The complete genome sequence of herpesvirus papio 2 (Cercopithecine herpesvirus 16) shows evidence of recombination events among various progenitor herpesviruses. J Virol (2006) 80:1214–21. doi: 10.1128/JVI.80.3.1214-1221.2006 PMC1346941 16414998

[46] Haanstra KG Dijkman K Bashir N Bauer J Mary C Poirier N. Selective blockade of CD28-mediated T cell costimulation protects rhesus monkeys against acute fatal experimental autoimmune encephalomyelitis. J Immunol (2015) 194:1454–66. doi: 10.4049/jimmunol.1402563 25589073

[47] Haanstra KG Wubben JAM Jonker M Hart BA‘t. Induction of encephalitis in rhesus monkeys infused with lymphocryptovirus-infected b-cells presenting MOG(34-56) peptide. PloS One (2013) 8:e71549. doi: 10.1371/journal.pone.0071549 23977076 PMC3744571

[48] Estep RD Hansen SG Rogers KS Axthelm MK Wong SW. Genomic characterization of Japanese macaque rhadinovirus, a novel herpesvirus isolated from a nonhuman primate with a spontaneous inflammatory demyelinating disease. J Virol (2013) 87:512–23. doi: 10.1128/JVI.02194-12 PMC3536378 23097433

[49] Axthelm MK Bourdette DN Marracci GH Su W Mullaney ET Manoharan M. Japanese Macaque encephalomyelitis: a spontaneous multiple sclerosis-like disease in a nonhuman primate. Ann Neurol (2011) 70:362–73. doi: 10.1002/ana.22449 PMC3170450 21674589

[50] Govindan AN Fitzpatrick KS Manoharan M Tagge I Kohama SG Ferguson B. Myelin-specific T cells in animals with Japanese macaque encephalomyelitis. Ann Clin Transl Neurol (2021) 8:456–70. doi: 10.1002/acn3.51303 PMC7886046 33440071

[51] Blair TC Manoharan M Rawlings-Rhea SD Tagge I Kohama SG Hollister-Smith J. Immunopathology of Japanese macaque encephalomyelitis is similar to multiple sclerosis. J Neuroimmunol (2016) 291:1–10. doi: 10.1016/j.jneuroim.2015.11.026 26857488 PMC4748211

[52] Kanai K Takashima K Okuno K Kato K Sano H Kuwamoto S. Lifelong persistent EBV infection of rabbits with EBER1-positive lymphocyte infiltration and mild sublethal hemophagocytosis. Virus Res (2010) 153:172–8. doi: 10.1016/j.virusres.2010.07.026 20691737

[53] Khan G Ahmed W Philip PS Ali MH Adem A. Healthy rabbits are susceptible to Epstein-Barr virus infection and infected cells proliferate in immunosuppressed animals. Virol J (2015) 12:28. doi: 10.1186/s12985-015-0260-1 25851649 PMC4340116

[54] Okuno K Takashima K Kanai K Ohashi M Hyuga R Sugihara H. Epstein-Barr Virus can infect rabbits by the intranasal or peroral route: an animal model for natural primary EBV infection in humans. J Med Virol (2010) 82:977–86. doi: 10.1002/jmv.21597 20419811

[55] Reguraman N Hassani A Philip P Khan G. Uncovering early events in primary Epstein-Barr virus infection using a rabbit model. Sci Rep (2021) 11:21220. doi: 10.1038/s41598-021-00668-x 34707156 PMC8551192

[56] Hassani A Reguraman N Shehab S Khan G. Primary peripheral Epstein-Barr virus infection can lead to CNS infection and neuroinflammation in a rabbit model: Implications for multiple sclerosis pathogenesis. Front Immunol (2021) 12:764937. doi: 10.3389/fimmu.2021.764937 34899715 PMC8656284

[57] Takashima K Ohashi M Kitamura Y Ando K Nagashima K Sugihara H. A new animal model for primary and persistent Epstein-Barr virus infection: human EBV-infected rabbit characteristics determined using sequential imaging and pathological analysis. J Med Virol (2008) 80:455–66. doi: 10.1002/jmv.21102 18205213

[58] Simas JP Efstathiou S. Murine gammaherpesvirus 68: a model for the study of gammaherpesvirus pathogenesis. Trends Microbiol (1998) 6:276–82. doi: 10.1016/s0966-842x(98)01306-7 9717216

[59] Weck KE Kim SS Virgin HW IV Speck SH. Macrophages are the major reservoir of latent murine gammaherpesvirus 68 in peritoneal cells. J Virol (1999) 73:3273–83. doi: 10.1128/JVI.73.4.3273-3283.1999 PMC104091 10074181

[60] Flaño E Kim I-J Moore J Woodland DL Blackman MA. Differential gamma-herpesvirus distribution in distinct anatomical locations and cell subsets during persistent infection in mice. J Immunol (2003) 170:3828–34. doi: 10.4049/jimmunol.170.7.3828 12646650

[61] Canny SP Goel G Reese TA Zhang X Xavier R Virgin HW. Latent gammaherpesvirus 68 infection induces distinct transcriptional changes in different organs. J Virol (2014) 88:730–8. doi: 10.1128/JVI.02708-13 PMC3911696 24155394

[62] Cardin RD Brooks JW Sarawar SR Doherty PC. Progressive loss of CD8+ T cell-mediated control of a gamma-herpesvirus in the absence of CD4+ T cells. J Exp Med (1996) 184:863–71. doi: 10.1084/jem.184.3.863 PMC2192775 9064346

[63] Brooks JW Hamilton-Easton AM Christensen JP Cardin RD Hardy CL Doherty PC. Requirement for CD40 ligand, CD4(+) T cells, and b cells in an infectious mononucleosis-like syndrome. J Virol (1999) 73:9650–4. doi: 10.1128/JVI.73.11.9650-9654.1999 PMC113004 10516078

[64] Casiraghi C Citlali Márquez A Shanina I Steven Horwitz M. Latent virus infection upregulates CD40 expression facilitating enhanced autoimmunity in a model of multiple sclerosis. Sci Rep (2015) 5:13995. doi: 10.1038/srep13995 26356194 PMC4564856

[65] Jog NR McClain MT Heinlen LD Gross T Towner R Guthridge JM. Epstein Barr Virus nuclear antigen 1 (EBNA-1) peptides recognized by adult multiple sclerosis patient sera induce neurologic symptoms in a murine model. J Autoimmun (2020) 106:102332. doi: 10.1016/j.jaut.2019.102332 31515129 PMC6930324

[66] Lanz TV Brewer RC Ho PP Moon J-S Jude KM Fernandez D. Clonally expanded b cells in multiple sclerosis bind EBV EBNA1 and GlialCAM. Nature (2022) 603:321–7. doi: 10.1038/s41586-022-04432-7 PMC9382663 35073561

[67] Polepole P Bartenslager A Liu Y Petro TM Fernando S Zhang L. Epstein Barr Virus-immortalized Blymphocytes exacerbate experimental autoimmune encephalomyelitis in xenograft mice. J Med Virol (2021) 93:3813–23. doi: 10.1002/jmv.26188 PMC7738365 32543727

[68] Chen H Zhong L Zhang W Zhang S Hong J Zhou X. Dose-dependent outcome of EBV infection of humanized mice based on green raji unit (GRU) doses. Viruses (2021) 13:2184. doi: 10.3390/v13112184 34834989 PMC8624110

[69] Volk V Theobald SJ Danisch S Khailaie S Kalbarczyk M Schneider A. PD-1 blockade aggravates Epstein-Barr virus+ post-transplant lymphoproliferative disorder in humanized mice resulting in central nervous system involvement and CD4+ T cell dysregulations. Front Oncol (2020) 10:614876. doi: 10.3389/fonc.2020.614876 33511078 PMC7837057

[70] Nielsen TR Rostgaard K Askling J Steffensen R Oturai A Jersild C. Effects of infectious mononucleosis and HLA-DRB1*15 in multiple sclerosis. Mult Scler (2009) 15:431–6. doi: 10.1177/1352458508100037 19153174

[71] Soldan SS Su C Lamontagne RJ Grams N Lu F Zhang Y. Epigenetic plasticity enables CNS-trafficking of EBV-infected b lymphocytes. PloS Pathog (2021) 17:e1009618. doi: 10.1371/journal.ppat.1009618 34106998 PMC8216538

[72] Jagessar SA Fagrouch Z Heijmans N Bauer J Laman JD Oh L. The different clinical effects of anti-BLyS, anti-APRIL and anti-CD20 antibodies point at a critical pathogenic role of γ-herpesvirus infected b cells in the marmoset EAE model. J Neuroimmune Pharmacol (2013) 8:727–38. doi: 10.1007/s11481-013-9448-6 23508625

[73] Jagessar SA Holtman IR Hofman S Morandi E Heijmans N Laman JD. Lymphocryptovirus infection of nonhuman primate b cells converts destructive into productive processing of the pathogenic CD8 T cell epitope in myelin oligodendrocyte glycoprotein. J Immunol (2016) 197:1074–88. doi: 10.4049/jimmunol.1600124 PMC4974490 27412414

[74] Hart BA‘t. A tolerogenic role of cathepsin G in a primate model of multiple sclerosis: Abrogation by Epstein-Barr virus infection. Arch Immunol Ther Exp (Warsz) (2020) 68:21. doi: 10.1007/s00005-020-00587-1 32556812 PMC7299916

[75] Skalsky RL Barr SA Jeffery AJ Blair T Estep R Wong SW. Japanese Macaque rhadinovirus encodes a viral MicroRNA mimic of the miR-17 family. J Virol (2016) 90:9350–63. doi: 10.1128/JVI.01123-16 PMC5044857 27512057

[76] Osborne AJ Atkins HM Balogh KK Brendle SA Shearer DA Hu J. Antibody-mediated immune subset depletion modulates the immune response in a rabbit (Oryctolagus cuniculus) model of Epstein-Barr virus infection. Comp Med (2020) 70:312–22. doi: 10.30802/AALAS-CM-20-000019 PMC7574219 32972486

[77] Meier U-C Giovannoni G Tzartos JS Khan G. Translational mini-review series on b cell subsets in disease. b cells in multiple sclerosis: drivers of disease pathogenesis and Trojan horse for Epstein-Barr virus entry to the central nervous system? Clin Exp Immunol (2012) 167:1–6. doi: 10.1111/j.1365-2249.2011.04446.x 22132878 PMC3248080

[78] Frischer JM Weigand SD Guo Y Kale N Parisi JE Pirko I. Clinical and pathological insights into the dynamic nature of the white matter multiple sclerosis plaque. Ann Neurol (2015) 78:710–21. doi: 10.1002/ana.24497 PMC4623970 26239536

[79] Cortese I Reich DS Nath A. Progressive multifocal leukoencephalopathy and the spectrum of JC virus-related disease. Nat Rev Neurol (2021) 17:37–51. doi: 10.1038/s41582-020-00427-y 33219338 PMC7678594

[80] Serafini B Rosicarelli B Magliozzi R Stigliano E Aloisi F. Detection of ectopic b-cell follicles with germinal centers in the meninges of patients with secondary progressive multiple sclerosis. Brain Pathol (2004) 14:164–74. doi: 10.1111/j.1750-3639.2004.tb00049.x PMC8095922 15193029

[81] Peters A Pitcher LA Sullivan JM Mitsdoerffer M Acton SE Franz B. Th17 cells induce ectopic lymphoid follicles in central nervous system tissue inflammation. Immunity (2011) 35:986–96. doi: 10.1016/j.immuni.2011.10.015 PMC3422678 22177922

[82] Wu Y Maruo S Yajima M Kanda T Takada K. Epstein-Barr Virus (EBV)-encoded RNA 2 (EBER2) but not EBER1 plays a critical role in EBV-induced b-cell growth transformation. J Virol (2007) 81:11236–45. doi: 10.1128/JVI.00579-07 PMC2045559 17686859

[83] Mauray S Fuzzati-Armentero MT Trouillet P Rüegg M Nicoloso G Hart M. Epstein-Barr Virus-dependent lymphoproliferative disease: critical role of IL-6. Eur J Immunol (2000) 30:2065–73. doi: 10.1002/1521-4141(200007)30:7<2065::AID-IMMU2065>3.0.CO;2-W 10940896

[84] Daniels BP Holman DW Cruz-Orengo L Jujjavarapu H Durrant DM Klein RS. Viral pathogen-associated molecular patterns regulate blood-brain barrier integrity *via* competing innate cytokine signals. mBio (2014) 5:e01476–01414. doi: 10.1128/mBio.01476-14 PMC4173776 25161189

[85] Förster C Burek M Romero IA Weksler B Couraud P-O Drenckhahn D. Differential effects of hydrocortisone and TNFα on tight junction proteins in an *in vitro* model of the human blood–brain barrier. J Physiol (2008) 586:1937–49. doi: 10.1113/jphysiol.2007.146852 PMC2375735 18258663

[86] Phares TW Kean RB Mikheeva T Hooper DC. Regional differences in blood-brain barrier permeability changes and inflammation in the apathogenic clearance of virus from the central nervous system. J Immunol (2006) 176:7666–75. doi: 10.4049/jimmunol.176.12.7666 16751414

[87] Tsao N Hsu HP Wu CM Liu CC Lei HY. Tumour necrosis factor-alpha causes an increase in blood-brain barrier permeability during sepsis. J Med Microbiol (2001) 50:812–21. doi: 10.1099/0022-1317-50-9-812 11549183

[88] Wong D Dorovini-Zis K Vincent SR. Cytokines, nitric oxide, and cGMP modulate the permeability of an *in vitro* model of the human blood-brain barrier. Exp Neurol (2004) 190:446–55. doi: 10.1016/j.expneurol.2004.08.008 15530883

[89] Bonney S Seitz S Ryan CA Jones KL Clarke P Tyler KL. Gamma interferon alters junctional integrity *via* rho kinase, resulting in blood-brain barrier leakage in experimental viral encephalitis. mBio (2019) 10:e01675–19. doi: 10.1128/mBio.01675-19 PMC6686045 31387911

[90] Chai Q He WQ Zhou M Lu H Fu ZF. Enhancement of blood-brain barrier permeability and reduction of tight junction protein expression are modulated by chemokines/cytokines induced by rabies virus infection. J Virol (2014) 88:4698–710. doi: 10.1128/JVI.03149-13 PMC3993813 24522913

[91] Li F Wang Y Yu L Cao S Wang K Yuan J. Viral infection of the central nervous system and neuroinflammation precede blood-brain barrier disruption during Japanese encephalitis virus infection. J Virol (2015) 89:5602–14. doi: 10.1128/JVI.00143-15 PMC4442524 25762733

[92] Minagar A Long A Ma T Jackson TH Kelley RE Ostanin DV. Interferon (IFN)-beta 1a and IFN-beta 1b block IFN-gamma-induced disintegration of endothelial junction integrity and barrier. Endothelium (2003) 10:299–307. doi: 10.1080/10623320390272299 14741845

[93] Cain MD Salimi H Gong Y Yang L Hamilton SL Heffernan JR. Virus entry and replication in the brain precedes blood-brain barrier disruption during intranasal alphavirus infection. J Neuroimmunol (2017) 308:118–30. doi: 10.1016/j.jneuroim.2017.04.008 PMC5694394 28501330

[94] Robinson WH Steinman L. Epstein-Barr Virus and multiple sclerosis. Science (2022) 375:264–5. doi: 10.1126/science.abm7930 35025606

[95] Pender MP Csurhes PA Smith C Douglas NL Neller MA Matthews KK. Epstein-Barr Virus-specific T cell therapy for progressive multiple sclerosis. JCI Insight (2018) 3:e124714. doi: 10.1172/jci.insight.124714 30429369 PMC6302936

[96] Serafini B Rosicarelli B Franciotta D Magliozzi R Reynolds R Cinque P. Dysregulated Epstein-Barr virus infection in the multiple sclerosis brain. J Exp Med (2007) 204:2899–912. doi: 10.1084/jem.20071030 PMC2118531 17984305

[97] Tzartos JS Khan G Vossenkamper A Cruz-Sadaba M Lonardi S Sefia E. Association of innate immune activation with latent Epstein-Barr virus in active MS lesions. Neurology (2012) 78:15–23. doi: 10.1212/WNL.0b013e31823ed057 22156987

[98] Lassmann H Niedobitek G Aloisi F Middeldorp JM NeuroproMiSe EBV Working Group. Epstein-Barr Virus in the multiple sclerosis brain: a controversial issue–report on a focused workshop held in the centre for brain research of the medical university of Vienna, Austria. Brain (2011) 134:2772–86. doi: 10.1093/brain/awr197 PMC3170536 21846731

[99] Hassani A Corboy JR Al-Salam S Khan G. Epstein-Barr Virus is present in the brain of most cases of multiple sclerosis and may engage more than just b cells. PloS One (2018) 13:e0192109. doi: 10.1371/journal.pone.0192109 29394264 PMC5796799

